# Bioavailable Dissolved Organic Carbon Serves as a Key Regulator of Phosphorus Dynamics in Stream Biofilms

**DOI:** 10.1111/1758-2229.70115

**Published:** 2025-06-01

**Authors:** Nuria Perujo, Daniel Graeber, Patrick Fink, Lola Neuert, Nergui Sunjidmaa, Markus Weitere

**Affiliations:** ^1^ Department of River Ecology Helmholtz Centre for Environmental Research–UFZ Magdeburg Germany; ^2^ Department of Aquatic Ecosystem Analysis Helmholtz Centre for Environmental Research − UFZ Magdeburg Germany

**Keywords:** bioavailable dissolved organic carbon, eutrophication, extracellular enzyme activity, microbial biofilms, phosphorus, sediments, sediment–water interface

## Abstract

Phosphorus (P) dynamics at the sediment–water interface of aquatic ecosystems are receiving increasing attention due to their implications for water quality. P uptake by microbial biofilms can serve as a mechanism to control and mitigate the risk of eutrophication. Microbial biofilms capture P both intracellularly and extracellularly. While the significance of extracellular P entrapment in biofilms in engineered systems has recently been established, little is known about its dynamics in aquatic ecosystems. Current research on eutrophication control predominantly emphasises nitrogen, phosphorus or nitrogen‐phosphorus ratio‐based approaches, often overlooking the potential indirect influence of bioavailable dissolved organic carbon (DOC) on P uptake by heterotrophic microorganisms. In this study, we tested the effect of bioavailable DOC on P entrapment patterns in biofilms and in biofilm P‐regulation mechanisms such as polyphosphate accumulation and alkaline phosphatase activity in semi‐natural flow‐through experimental flumes. Our results show that intracellular P entrapment is limited by bioavailable DOC, while extracellular P entrapment is independent of bioavailable DOC and has the potential to offset intracellular P saturation. We further demonstrate that DOC bioavailability influences benthic P cycling and that its implications extend into critical areas of ecosystem functioning such as river self‐purification, competitive resource utilisation and organic P cycling.

## Graphical abstract

We tested the effect of bioavailable DOC on P entrapment in biofilms and in biofilm P‐regulation mechanisms such as polyphosphate accumulation and alkaline phosphatase activity in semi‐natural experimental flumes. Our results show that intracellular P entrapment is limited by bioavailable DOC, while extracellular P entrapment is independent of bioavailable DOC and potential to offset intracellular P saturation. The implications of these results extend into critical areas of ecosystem functioning such as river self‐purification, competitive resource utilisation and organic P cycling.

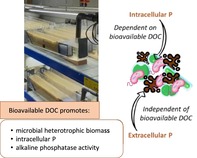

**Abbreviations**
APA: alkaline phosphatase activity
DOC: dissolved organic carbon
EPS: extracellular polymeric substances
N: nitrogen
P: phosphorus
TDP: total dissolved phosphorus

## 1 Introduction

In aquatic ecosystems, nutrient cycling is a crucial ecosystem function. Both nitrogen (N) and phosphorus (P) are essential nutrients for aquatic biota, and often P is the limiting nutrient for biomass growth in freshwaters (Correll 1999). In excess, N and P are a global ecosystem impairment, whereby excess nutrients modify the structure and function of freshwater ecosystems, causing eutrophication (McCall et al. 2017). Main impacts of eutrophication are an excessive increase in algal biomass and productivity; impairment of water physicochemical quality (i.e., increase in colour, odour and turbidity); anoxic waters, fish death and water use restriction for recreational purposes (Leaf 2018). Eutrophication has been recognised as a significant environmental concern across Europe since the late 1980s and continues to be a challenge nowadays (Bol et al. 2018). Current research on eutrophication control predominantly emphasises approaches controlling N, P or the molar N:P ratio (e.g., Graeber et al. 2024), with much less focus on the potential indirect influence of labile dissolved organic carbon (DOC) on N and P cycling (Li et al. 2024). Bioavailable DOC has been related to bacterial uptake of P for decades (Kirchman 1994), and it is well known that heterotrophic bacteria may account for more than 60% of P uptake in both freshwater and marine environments (Kirchman 1994). Also, it is known that the bacteria‐algae relationship plays a key role in the occurrence of algal blooms (Li et al. 2024). The heterotrophic component might be crucial for the systems' total P uptake and capacity, and a simple microcosm experiment with planktonic bacteria demonstrated that it is centrally controlled by the bioavailable DOC:P (Graeber et al. 2021).

Benthic communities develop in riverbed substrates forming consortia (biofilms) of autotrophic and heterotrophic microorganisms embedded in an extracellular polymeric substance (EPS) matrix (Lock et al. 1984). Biofilms are highly efficient ecological communities which play a crucial role in the biogeochemical cycles in sediments (Mermillod‐Blondin et al. 2005) and catalyse ecological processes such as mineralization of organic matter, assimilation of inorganic nutrients, as well as nutrient storage and transformation (Findlay et al. 2003). Perujo et al. (2018) found that nutrient accumulation in benthic biofilms plays a key role in DOC and total dissolved phosphorus (TDP) removal (up to 50% of removal efficiency) in sand filtration systems. The storage of P in biofilms can thereby contribute significantly to the reduction of P discharge from rivers (Wu et al. 2018). Benthic biofilms can accumulate P through intracellular and extracellular pathways (Xu, Ou, et al. 2020; Xu, Wu, et al. 2020). Extracellular P entrapment relies on P adsorption through electrostatic attraction, ligand exchange, physical adsorption, and ion exchange (Tan et al. 2023). The EPS matrix is a complex mixture of macromolecules, primarily composed of polysaccharides, but also containing various amounts of protein, lipid, DNA, and vitamins (Flemming and Wingender 2010) and may account for 50 to 90% of the total organic carbon of biofilms (Flemming 2016). Apart from nutrient storage, its main functions are to maintain the structural stability of biofilm and to protect it against environmental stress (Vu et al. 2009). The amount of P stored in EPS can account for as much as 40% of the total P retention in granular sludge reactors (Zhang et al. 2020). Also, the content of P in EPS accounted for a high proportion of the total P content in cyanobacteria aggregates (Li et al. 2024). Although the role of EPS in nutrient acquisition due to their adsorptive properties has been reported in the literature for a long time (e.g., Freeman and Lock 1995), only a few works consider the EPS matrix when studying P uptake in river ecosystems.

The patterns of intracellular and extracellular P entrapment in fluvial aquatic ecosystems are still unknown. Within‐biofilm P processes have been mainly studied in engineered systems such as microalgae accumulating polyphosphate granules (Slocombe et al. 2020) and polyphosphate accumulating organisms biofilm sequence batch reactors (Zhang et al. 2020). Unlike engineered systems, biofilms in aquatic ecosystems are mixed autotrophic and heterotrophic microbial communities which coexist under environmental variable conditions (e.g., along the river longitudinal gradient of light and dissolved organic matter quality) and not under fixed controlled parameters as is the case of engineered systems. Within the biofilm as a whole, both polyphosphates and the alkaline phosphatase activity (APA) are important components of the P regulation mechanisms. Polyphosphate accumulation is a biofilm P storage mechanism (Powell et al. 2009) in both autotrophic and heterotrophic microorganisms (Slocombe et al. 2020) and thus may modify P entrapment pathways. However, no clear trends have been found between polyphosphate occurrence and P uptake dynamics in stream biofilms (Saia et al. 2017). APA is responsible for mineralising organic P compounds to inorganic compounds that can be later assimilated in the biofilm biomass (Chróst and Krambeck 1986). APA is a bio‐indicator of low P conditions, activated at low ambient phosphate levels (e.g., Sanz‐Luque et al. 2020; Li and Dittrich 2019). All this opens up a new focus of study aimed at understanding the biological pathways within the P retention in benthic biofilms. Understanding biofilms as important drivers of P entrapment in river ecosystems forces us to consider (1) not only the autotrophic component but also the—oftentimes ignored—heterotrophic component (Ardón et al. 2021), (2) the extracellular P entrapment in biofilms, which increases its prevalence under high dissolved P concentrations (Perujo et al. 2024), and (3) the role of bioavailable DOC in biofilm P entrapment.

The main aim of this work is to test for the effect of bioavailable DOC on P entrapment patterns in biofilms and to assess the biofilm related mechanisms behind these effects. DOC in the studied stream is of low bioavailability (Pasqualini et al. 2024), hence resulting in severe DOC and P co‐limitation (Weitere et al. 2021) favouring algal biomass over heterotrophic ones. Our hypotheses are that, (i) addition of bioavailable DOC at molar C:P ratios > 100 will increase P entrapment in biofilms due to two different mechanisms depending on TDP concentrations: (ii) at low TDP concentrations, bioavailable DOC will promote intracellular P entrapment due to an increase in the microbial heterotrophic component which would decrease biofilm C:P molar ratio, increasing its P entrapment efficiency; (iii) at high TDP concentrations, the positive effects of bioavailable DOC on intracellular P entrapment will decrease but these will be compensated by high extracellular P entrapment due to higher EPS production. Overall, bioavailable DOC will promote P entrapment in fluvial biofilms.

## 2 Experimental Procedures

### 2.1 Experimental Design

The experimental design consisted of 10 flow‐through experimental flumes installed as a river water bypass system within a mobile mesocosm infrastructure (MOBICOS, see Fink et al. 2020 for details). The bypass experiment was located in the upper reach of the Holtemme River, a 3rd order stream in Central Germany (see Weitere et al. 2021 for details on its geological, physico‐chemical and toxicological background). River water was continuously pumped into each of the flumes at a rate of 2.8 mL/s. Physico‐chemical characteristics of the river water during the entire duration of the experiment were 10.6 ± 0.7 mg O_2_/L, 12°C ± 2.8°C, 117.8 ± 6.3 μS/cm^2^ and pH 7.5 ± 0.15. Mean nutrient concentrations in the river water were 12.3 ± 1.4 mg DOC/L, 3.8 ± 1.25 μg P/L, 1.26 ± 0.27 mg N‐NO_3_/L and 0.016 ± 0.004 mg N‐NH_4_/L. Each flume (72 cm length, 14 cm height, 8 cm width) was filled with a 10 cm deep layer of pre‐cleaned sand (0.8–1 mm grain size). The depth of the water column was 2 cm. Light conditions (14/10 h light: dark) inside MOBICOS were set to 108.39 ± 11.01 μmol photons m^2^/s of PAR with LED panels (SolarStinger SunStrip daylight; Econlux, Cologne, Germany) to allow biofilm colonisation and growth.

The experiment had a total duration of 21 days divided into two periods: a phase of biofilm establishment under different P treatments (“*before*”) and a phase of DOC manipulation in addition to the P manipulation (“*after*”). In each one of these periods, there were 3 control flumes which consisted of untreated river water that did not receive any additional P or DOC input (before control [BC] and after control [AC], Table 1). For a total of 2 weeks in the *before* phase, each flume other than the controls continuously received a different P concentration following a gradient of TDP concentrations from 8 to 445 μg P/L (before impact [BI], 7 flumes, Table 1). TDP concentrations in the flumes were adjusted by continuously adding a concentrated KH_2_PO_4_ solution using a peristaltic pump. For a total of 1 week in the *after* phase, each flume other than the controls maintained the gradient of TDP concentrations, along with labile C added from a dextrose monohydrate preparation resulting in a C_labile_:P molar ratio of 409 for the supplemented DOC and P in all the flumes (after impact [AI], 7 flumes, Table 1). TDP and DOC concentrations in the flumes were measured from filtered (0.2 μm nylon filter) water samples. TDP concentration was determined by ICP‐MS, previously acidified with 65% HNO_3_. DOC concentration was determined by TOC analyser, previously acidified with HCl.

**TABLE 1:** Treatments tested and their description.

Flume no.	Before (P gradient, low DOC bioavailability)		After (P gradient, high DOC bioavailability)	
	Treatment code	Description	Treatment code	Description
1	BC	Control	AC	Control
2	BC	Control	AC	Control
3	BC	Control	AC	Control
4	BI	TDP 30	AI	TDP 30 + DOC
5	BI	TDP 55	AI	TDP 55 + DOC
6	BI	TDP 70	AI	TDP 70 + DOC
7	BI	TDP 120	AI	TDP 120 + DOC
8	BI	TDP 135	AI	TDP 135 + DOC
9	BI	TDP 165	AI	TDP 165 + DOC
10	BI	TDP 300	AI	TDP 300 + DOC

Abbreviations: AC: after‐control; AI: after‐impact; BC: before‐control; BI: before‐impact.

To examine the response in the *before* phase, we sampled the biofilm in sediment at two times: 7 and 14 days after initiating P addition. We then calculated the average. Sampling at these two time‐points during the *before* phase ensures that the biofilm is already in the mature stage rather than the growing stage, facilitating a meaningful comparison between the *before* and *after* phases. To analyse the response in the *after* phase, we sampled the biofilm in sediment at the conclusion of the experiment (21 days after the experiment began, 7 days after initiating the *P* + DOC addition).

### 2.2 Biofilm Analyses

#### 2.2.1 Biofilm Structural Components: Chlorophyll—A (*Chl‐a*), EPS and Bacterial Density

Samples for *Chl‐a* analysis were placed in plastic vials and kept in the dark at (−20°C) until analysis. *Chl‐a* concentration was determined as described by Jeffrey and Humphrey (1975). Acetone 90% (10 mL) was added to each sediment sample in order to extract the *Chl‐a* and kept in the dark for 8–12 h at 4°C. Sediment samples were sonicated and filtered (GF/C, 1.4 μm, 47 mm). Absorbance was measured at 430, 665 and 750 nm. Results are given as μg of chlorophyll‐a/cm^3^.

EPS were extracted by a cation exchange resin (CER) and the content of polysaccharides was measured spectrophotometrically following the protocol described by DuBois et al. (1956) and detailed in Perujo et al. (2017). Briefly, sediment samples (1 cm^3^) were placed in a 2 mL microtube with phosphate buffer saline (PBS, 1 mL) and 0.3 g of CER. After shaking the microtubes carefully, samples were incubated in ice for 1 h in a shaker (250 rpm). Samples were then centrifuged (11,000 rpm) for 15 min at 4°C. The supernatant (500 μL) from each sample was pipetted into glass tubes. A phenol solution (12.5 μL, 80% w/w) was added to the glass tubes. After carefully shaking, 1.25 mL of H_2_SO_4_ (95.5%) was added to the samples. Glass tubes were capped. After 10 min, samples were carefully shaken and incubated for 20 min in a water bath (30°C). Absorbance (485 nm) was measured in a multiplate reader. To determine EPS concentration, a glucose standard was prepared. Results are given as μg glucose‐equivalents/cm^3^.

Bacterial density was determined by flow cytometry (Accuri C6 Flow cytometer) following a protocol adapted from Amalfitano et al. (2009). Sediment samples (1 cm^3^) were placed in a 20 mL autoclaved glass vial with detaching solution (5 mL, NaCl (130 mM), Na_2_HPO_4_ (7 mM), NaH_2_PO_4_ (3 mM), formaldehyde (37%), sodium pyrophosphate decahydrate 99% (0.1% final concentration) and tween 20 (0.5% final concentration)). Samples were vortexed and shaken in the dark at room temperature (30 min at 150–200 rpm), then cooled at 4°C for 10 min and sonicated with ice (2 cycles of 1 min, 30 s rest in‐between). Samples were vortexed for 8–10 s and the supernatant (1 mL) was pipetted into a sterile microtube. OptiPrep density gradient (1 mL) was added in the bottom of the microtube. Samples were centrifuged for 90 min (14,000 rpm at 4°C). The supernatant (2 mL) was pipetted into a new sterile microtube and vortexed. An aliquot (400 μL) of the sample was placed in a cytometer tube and stained with Syto 13 (4 μL, 5 μM solution). Samples were incubated in the dark for 15–30 min and then a bead solution (10 μL, solution 10^6^ beads/ml, 1.0 μm) was added as an internal standard. Bacterial density was measured in a flow cytometer. Results are given as bacteria cells × 10^6^/cm^3^.

#### 2.2.2 Biofilm Elemental Composition: C, N and P

Sediment biofilm samples (1 cm^3^) were placed in a 10 mL tube with Ringer solution (5 mL). To detach biofilm from sediments, samples were vortexed (10 s), sonicated in a 1‐min cycle with ice to avoid cell lysis (Amalfitano and Fazi 2008) and vortexed again (15 s). The supernatant (3 mL) of each sample was placed into a new tube and the total phosphorus of the biofilm extract analysed by ICP‐MS. Results are given as μg P/cm^3^. For carbon and nitrogen determination, aliquots of the extract (100 μL) were pipetted into pre‐weighed tin cups and placed in the oven at 60°C until dry. The remaining extract was kept at 4°C. Pipetting was repeated five times to obtain a final dry weight of ca. 1–3 mg. Tin cups were weighed and sediment carbon and nitrogen content were analysed in a CN‐analyser (Carlo Erba).

#### 2.2.3 Intracellular and Extracellular Phosphorus Entrapment in Biofilms

For P extra determination, extracts from EPS in the biofilms were prepared as follows, adapted from Perujo et al. (2017). Sediment biofilm samples (1 cm^3^) were placed in a 2 mL microcentrifugation tube with Ringer solution (1 mL) and 0.3 g of cationic exchange resin. Samples were incubated in ice for 1 h at 250 rpm. After incubation, samples were centrifuged (11,000 rpm−12,000 *g*) at 4°C for 15 min. The supernatant (500 μL) of each sample was placed in a new tube and the total phosphorus of the biofilm extracellular polymeric matrix extract analysed by ICP‐MS. Intracellular P in the biofilm (P intra) was calculated from the difference between total phosphorus in the biofilm extract (P biofilm) and the total phosphorus in the EPS (P extra). Results are given as μg P/cm^3^.

#### 2.2.4 Mechanisms of P Regulation in Biofilms: APA and Polyphosphate Accumulation

APA (EC 3.1.3.1) was measured following the protocol described in Perujo et al. (2017). Briefly, sediment biofilm samples (1 cm^3^) were placed in a 15 mL tube with Ringer solution (4 mL) and 120 μL of artificial substrate methylumbelliferyl (MUF)‐phosphate (saturating conditions, 0.3 mM, Romaní and Sabater (2000)). A substrate blank was prepared with milliQ water to determine the abiotic hydrolysis of the substrate itself. Samples and blanks were incubated for 1 h in the dark with agitation. After 1 h incubation, glycine buffer (4 mL, pH 10.4) was added to stop the reaction and maximise MUF fluorescence. Samples were centrifuged (2000 *g*) for 2 min, and the supernatant (350 μL) of each sample was placed into a 96‐well black plate. Fluorescence was measured at excitation/emission wavelengths of 365/455 in a fluorimeter plate reader (Tecan, Safire^2^). To determine extracellular enzyme activity, MUF standards were prepared. Results are given in μmol MUF/cm^3^·h.

For polyphosphate determination, sediment biofilm samples (1 cm^3^) were placed in a 10 mL tube with Ringer solution (5 mL). To detach biofilm from sediments, samples were vortexed (10 s), sonicated in a 1‐min cycle with ice to avoid cell lysis (Amalfitano and Fazi 2008) and vortexed again (15 s). The supernatant (1 mL) of each sample was stained with 2 μL 4′6‐diamidino‐2‐phenylindole (DAPI) (final concentration 10 μg/mL) for 30 min in the dark (Terashima et al. 2016). The supernatant (350 μL) of each sample was placed into a 96‐well black plate. Fluorescence was measured at excitation/emission wavelength of 355/535 (polyphosphate fluorescence, Tijssen et al. 1982) and at 355/450 (DNA fluorescence, Tijssen et al. 1982) in a fluorimeter plate reader (Tecan, Safire^2^). Results are given as a relative polyphosphate fluorescence values by normalising polyphosphate fluorescence with DNA fluorescence (Terashima et al. 2016).

### 2.3 Data Treatment

To test for the effect of DOC bioavailability on P entrapment in biofilms, data from biofilm samples was analysed using a two‐way repeated measures ANOVA to test for differences between *control‐impact* flumes and *before*–*after* treatments, with the error term to account for the within‐subject correlation. The overall effect of DOC availability was shown by the interaction between *before–after* and *control–impact*. We used the function *anova* in the {stats} package. Previous to the ANOVA test, variables were log‐transformed to meet ANOVA assumptions of data normality and homogeneity of variances. Different patterns along TDP concentrations were further assessed in *before* and *after* treatments, separately. To do so, we studied curve fitting in regression models using the function *geom_smooth* in the {ggplot2} package and adjusting to a quadratic model with a bootstrap of *n* = 1000 iterations and a confidence level of 0.95. To assess the fitting of the regression models, the estimates, the *t* values, and the *p* values of the intercept, the linear and the quadratic terms were checked using the function *tidy_model* in the {broom} package.

To visualise the overall effect of DOC bioavailability on P entrapment in biofilms, data from biofilm analyses was plotted together in a multivariate principal component analysis (PCA) followed by pairwise PERMANOVA comparisons to test the differences between the 4 treatments: BC, BI, AC and AI. To study how the linkage between biofilm structural components, elemental composition, intracellular and extracellular phosphorus entrapment, and mechanisms of P‐regulation are influenced by DOC bioavailability, we separately analysed the biofilm's multivariate response for the *before* and *after* phases in two PCAs. We used the functions *prcomp*, *fviz_pca* and *adonis2* in the {stats}, {factoextra} and {vegan} packages, respectively. Before the multivariate analysis, variables were log‐transformed and scaled to meet normality, stabilise variances and equalise the importance of variables. All statistical analyses were carried out with R (version 4.1.1).

## 3 Results

### 3.1 Overall Effect of Bioavailable DOC on Biofilm P‐Related Variables Along TDP Gradients

**FIGURE 1:**
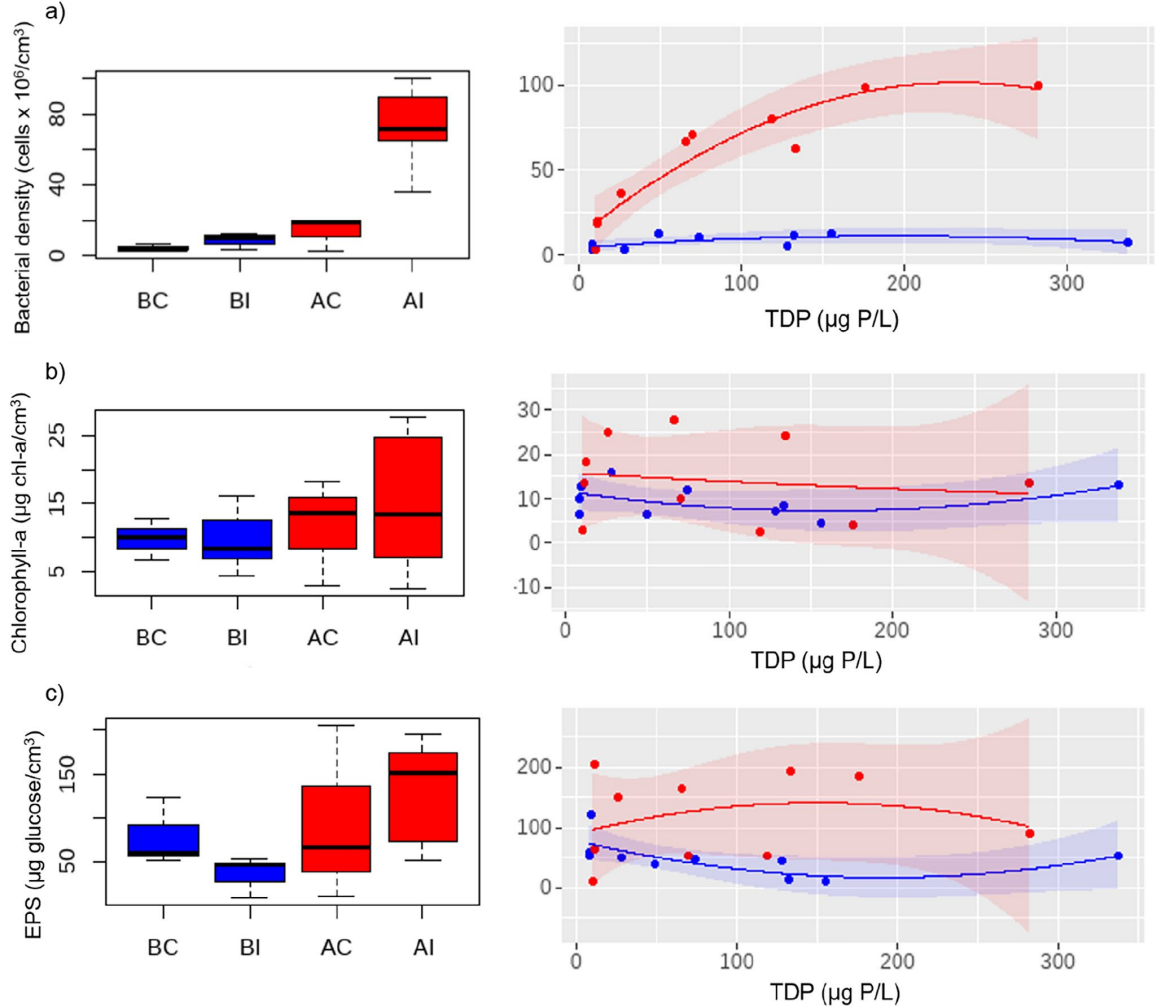
Biofilm structural variables. Left: Boxplots of the treatments BC: before‐control, BI: before‐impact (low DOC bioavailability), AC: after‐control and AI: after‐impact (high DOC bioavailability). Right: Curve fitting of control and impact treatments along the TDP gradient under the scenarios of low DOC bioavailability (before phase, blue) and high DOC bioavailability (after phase, red). The shaded area of the curves corresponds to a confidence level of 0.95. See Table S1 for the fitting of the regression models.

Bacterial density showed a significant interaction between *before–after* and *control–impact*, highlighting the influence of DOC bioavailability on bacterial density (*p* < 0.05, Table 2). Specifically, the bioavailability of DOC increased bacterial density from a median of 3.27, 9.96 and 18.52 bacteria cells x 10^6^/cm^3^ in BC, BI and AC, respectively, to 71.43 bacteria cells × 10^6^/cm^3^ when supplemented with bioavailable DOC (AI treatment, Figure 1a). This increase in bacterial density followed a quadratic regression with TDP in the presence of bioavailable DOC (red curve in Figure 1a, Table S1). Conversely, at low DOC bioavailability, bacterial density did not vary along the TDP gradient. There was no effect of DOC bioavailability on *Chl‐a* (Table 2, Figure 1b, Table S1) with median values of 10, 8.3, 13.6 and 13.4 μg *Chl‐a*/cm^3^ in BC, BI, AC and AI, respectively. Regarding EPS, the results did not show an interaction between the *before*–*after* and *control*–*impact* (n.s, Table 2). However, on closer examination, we saw that the EPS concentration decreased following a quadratic function along the TDP gradient under low DOC bioavailability (blue curve in Figure 1c, Table S1). Conversely, in situations of high DOC bioavailability, the EPS concentration did not show significant differences along the TDP gradient. The median values of EPS concentration were 60.4, 45.9, 66.3 and 151.4 μg glucose/cm^3^ in BC, BI, AC and AI, respectively.

**TABLE 2:** Results of the ANOVA two factor (*n* = 20).

	Before:after (BA)	Control:impact (CI)	BA:CI
Bacteria density	*p* < 0.05	*p* < 0.05	***p* < 0.05**
*Chl‐a*	n.s	n.s	n.s
EPS	*p* < 0.05	n.s	n.s
C biofilm	*p* < 0.05	*p* < 0.05	*p < 0.1*
N biofilm	*p* < 0.05	*p* < 0.05	*p < 0.1*
P biofilm	*p* < 0.05	*p* < 0.05	n.s
C:P biofilm	n.s	n.s	n.s
P intra	*p* < 0.05	n.s	n.s
P extra	*p < 0.1*	*p < 0.1*	n.s
Ratio P_intra_/P_extra_	*p* < 0.05	*p* < 0.05	***p* < 0.05**
APA	n.s	n.s	n.s
Polyphosphate (RFU)	*p* < 0.05	*p* < 0.05	n.s

*Note:* Before:after (BA), control:impact (CI) and the interaction BA:CI in the biofilm variables. Values indicate statistically significant different results with *p* < 0.05 and *p* < 0.1. Non‐significant results are expressed as n.s. Values in bold indicate statistically significant BA:CI interaction (*p* < 0.05). Values in italics indicate BA:CI interaction at a *p* level of 0.1.

**FIGURE 2:**
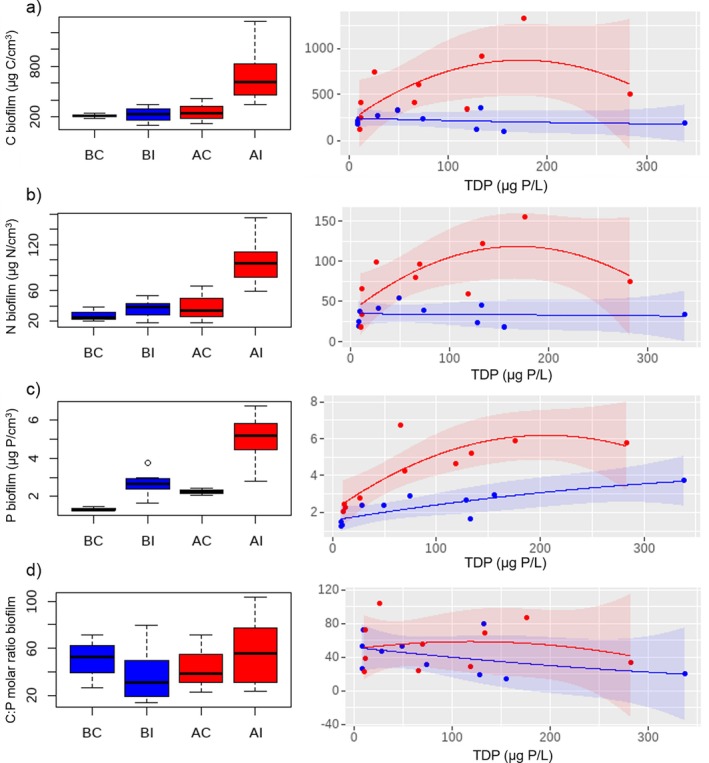
Biofilm elemental composition. Boxplots of the treatments BC: before‐control, BI: before‐impact (low DOC bioavailability), AC: after‐control and AI: after‐impact (high DOC bioavailability). Right: Curve fitting of control and impact treatments along the TDP gradient under the scenarios of low DOC bioavailability (before phase, blue) and high DOC bioavailability (after phase, red). The shaded area of the curves corresponds to a confidence level of 0.95. See Table S1 for the fitting of the regression models.

The elemental composition of the biofilm in terms of C and N showed a slightly significant interaction between *before–after* and *control–impact*, indicating an effect of DOC bioavailability on the content of C and N in the biofilm (Table 2, *p* < 0.1). C and N in the biofilm showed higher concentrations in the AI treatment. The median values were 214, 235, 246 and 611 μg C/cm^3^, and 25.1, 38.4, 33.6 and 95.9 μg N/cm^3^ for BC, BI, AC, AI, respectively (Figure 2a,b). At high DOC bioavailability, biofilm C and N increased along the TDP gradient following a quadratic regression (red curve, Figure 2a,b), although the regression coefficients showed only slight significance for biofilm N (Table S1). As for P in the biofilm, the median values were 1.3, 2.6, 2.3 and 5.2 μg P/cm^3^ for BC, BI, AC and AI, respectively, with no significant interaction *before*–*after* and *control*–*impact*. However, when examining it along the TDP gradient, P concentration in the biofilm showed a linear relationship under conditions of low DOC bioavailability (blue curve, Figure 2c), while under conditions of high DOC bioavailability, the relationship tended towards a quadratic fitting (red curve, Figure 2c) (Table S1). Results showed that there is no effect from the bioavailability of DOC on biofilm C:P molar ratios (Table 2, Figure 2d, Table S1) with median values of 52.8, 31.2, 38.9 and 55.7 in BC, BI, AC and AI, respectively.

**FIGURE 3:**
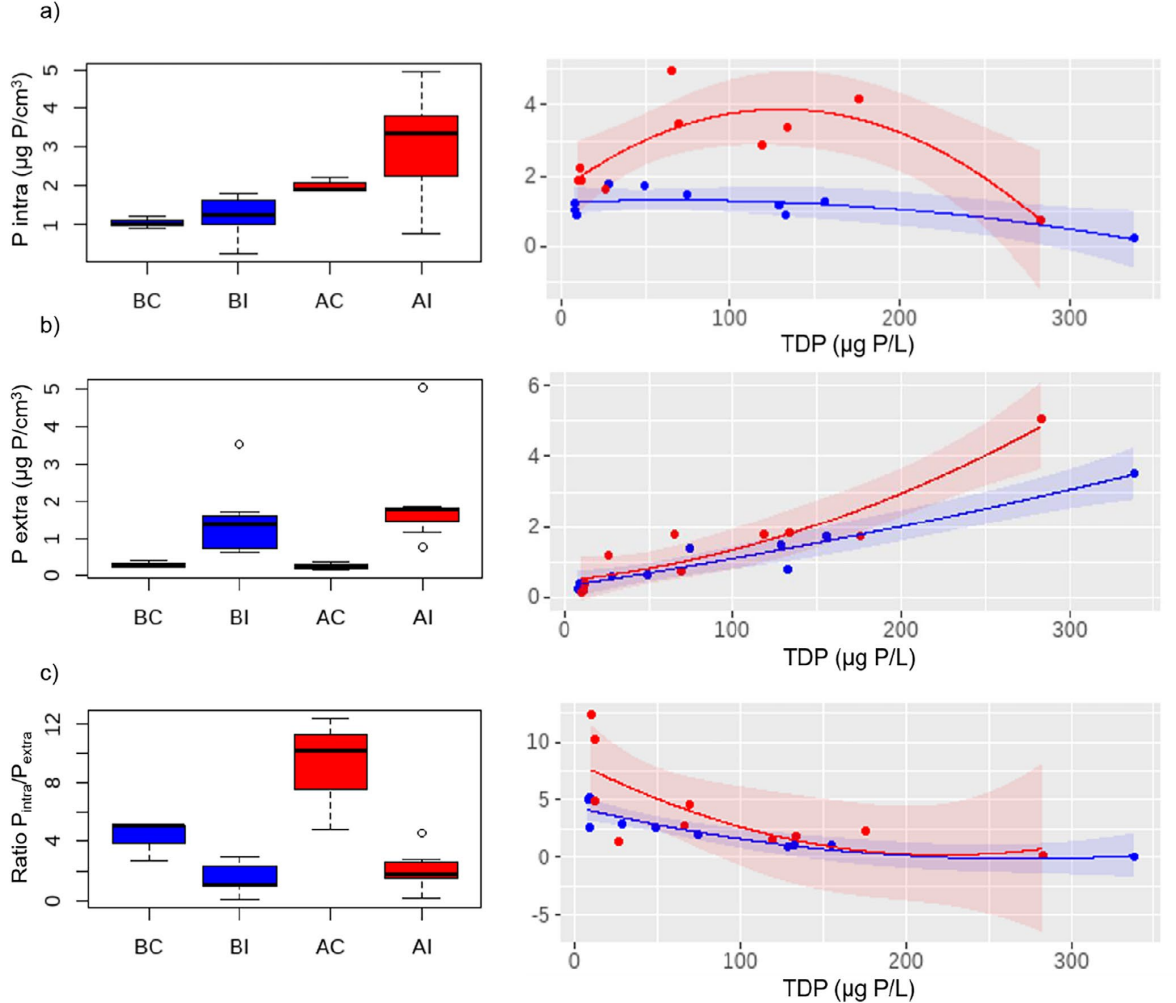
Intracellular and extracellular phosphorus entrapment in biofilms. Boxplots of the treatments BC: before‐control, BI: before‐impact (low DOC bioavailability), AC: after‐control and AI: after‐impact (high DOC bioavailability). Right: Curve fitting of control and impact treatments along the TDP gradient under the scenarios of low DOC bioavailability (before phase, blue) and high DOC bioavailability (after phase, red). The shaded area of the curves corresponds to a confidence level of 0.95. See Table S1 for the fitting of the regression models.

Intracellular and extracellular P entrapment in biofilms did not show significant interactions *before*–*after* and *control*–*impact* (Table 2). However, greater intracellular uptake occurred in the presence of bioavailable DOC (AI treatment, Figure 3a). The median values were 1.02, 1.26, 1.90 and 3.35 μg P/cm^3^ for BC, BI, AC and AI, respectively. Along the TDP gradient, intracellular P uptake changed depending on the presence of bioavailable DOC. Under low DOC bioavailability, intracellular P uptake decreased linearly as the TDP concentration increased (blue curve, Figure 3a, Table S1). Conversely, under conditions of high DOC bioavailability, intracellular P uptake increased and then decreased, following a quadratic regression along the TDP concentration (red curve, Figure 3a, Table S1). Extracellular P entrapment showed a similar response regardless of bioavailable DOC, with median values of 0.26, 1.37, 0.22 and 1.79 μg P/cm^3^ in BC, BI, AC and AI, respectively. Specifically, extracellular P entrapment increased following a linear relationship along the TDP concentration (Figure 3b, Table S1). The ratio P_intra_/P_extra_ showed a significant interaction *before*–*after* and *control*–*impact* (*p* < 0.05, Table 2). However, analysing the response along the TDP gradient, no significant differences were seen between high and low DOC bioavailability. Under both conditions, the P_intra_/P_extra_ ratio decreased along the TDP gradient following a linear regression (Figure 3c, Table S1). The median values were 5.1, 1.1, 10.2 and 1.8 for BC, BI, AC and AI, respectively.

**FIGURE 4:**
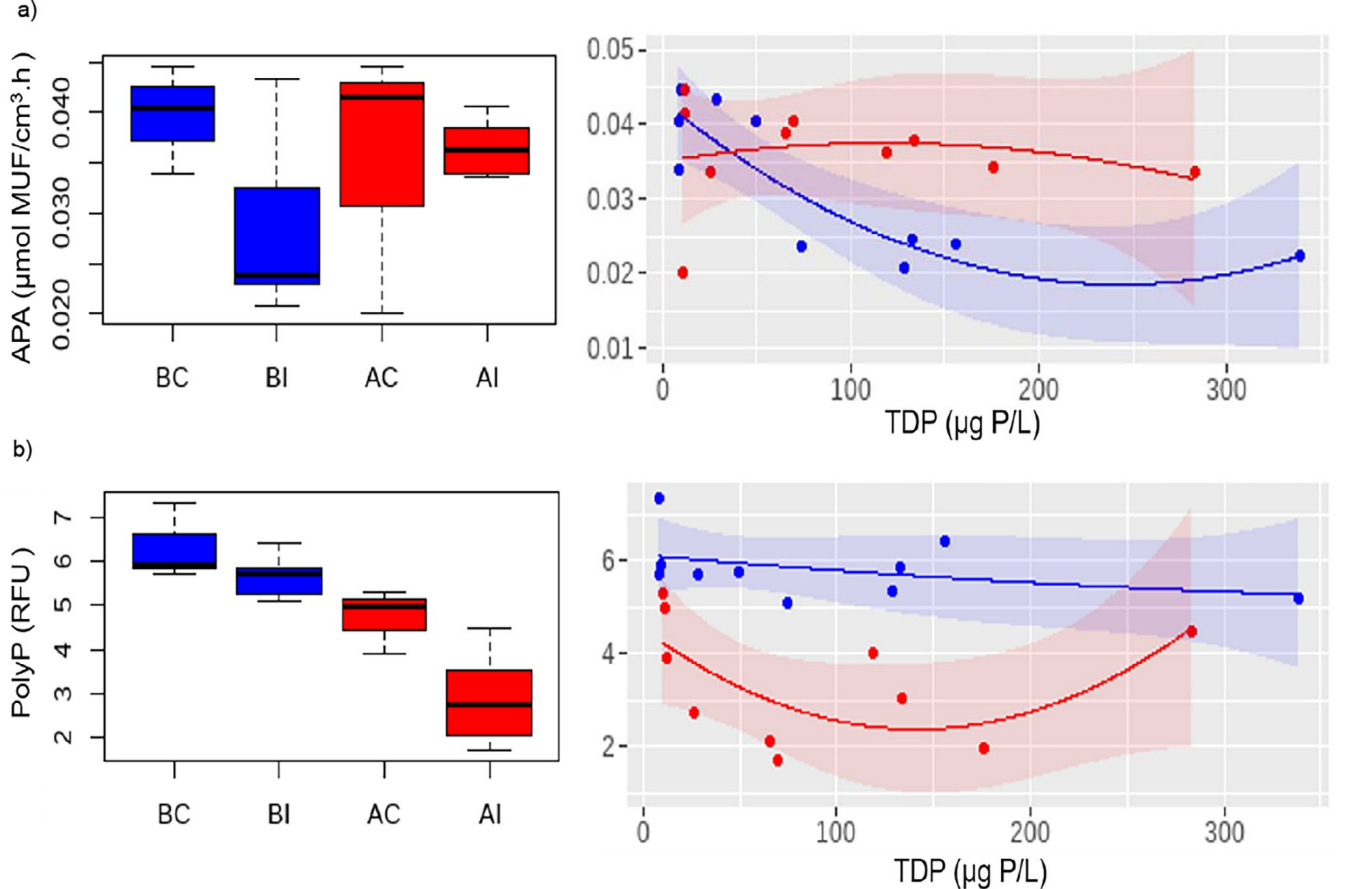
Mechanisms of P‐regulation in biofilms: Alkaline phosphatase enzyme activity and polyphosphate accumulation. Boxplots of the treatments BC: before‐control, BI: before‐impact (low DOC bioavailability), AC: after‐control and AI: after‐impact (high DOC bioavailability). Right: Curve fitting of control and impact treatments along the TDP gradient under the scenarios of low DOC bioavailability (before phase, blue) and high DOC bioavailability (after phase, red). The shaded area of the curves corresponds to a confidence level of 0.95. See Table S1 for the fitting of the regression models.

Mechanisms of P‐regulation in biofilms (e.g., APA and polyphosphate accumulation) did not show significant interactions *before*–*after* and *control*–*impact* (Table 2). However, when analysing the response along the TDP gradient, we observed a different response depending on DOC bioavailability. APA decreased as the TDP concentration increased, following a linear‐to‐quadratic relationship in situations of low DOC bioavailability (blue curve Figure 4a, Table S1). However, in situations of high DOC bioavailability, APA did not respond to TDP concentrations. Median values for APA were 0.040, 0.024, 0.041 and 0.036 nmol MUF/h·cm^3^ in BC, BI, AC and AI, respectively. As for the presence of polyphosphate, in situations of low DOC bioavailability, there was no clear response along the TDP gradient, but with high DOC bioavailability, the presence of polyphosphate decreased along the TDP gradient following a quadratic regression (red curve Figure 4b, Table S1). Median values for polyphosphate were 5.9, 5.7, 5.0 and 2.7 RFU in BC, BI, AC and AI, respectively.

**FIGURE 5:**
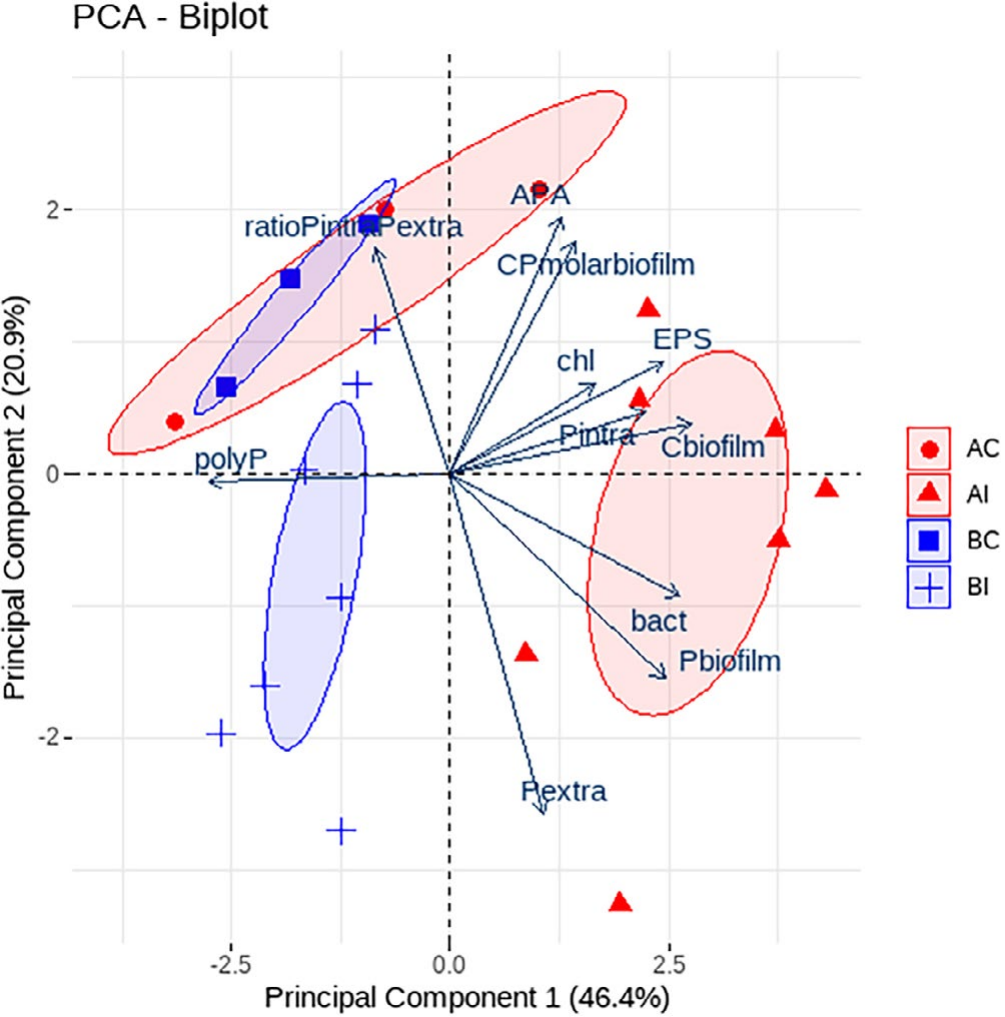
Principal component analysis (PCA) with the biofilm data from the 4 treatments: BC: before‐control, BI: before‐impact (low DOC bioavailability), AC: after‐control and AI: after‐impact (high DOC bioavailability). Ellipses are based on a 95% confidence interval for each treatment group. APA (alkaline phosphatase activity); chl (chlorophyll‐a); EPS (extracellular polymeric substances); Pintra (intracellular P); Cbiofilm (carbon content in the biofilm); bact (bacteria density); Pbiofilm (P content in the biofilm); Pextra (extracellular P); polyP (polyphosphates); CPmolarbiofilm (C:P molar ratio of the biofilm); ratioPintraPextra (ratio between intracellular P and extracellular P).

The multivariate PCA of all biofilm data explained a total variance of 67.3% in PCA 1 and 2 altogether (Figure 5). The PCA differentiated three groups based on a 95% confidence interval: control treatments (AC after‐control, and BC before‐control), BI (before‐impact, low bioavailable DOC), and AI (after‐impact, high bioavailable DOC). When performing pairwise PERMANOVA comparisons, significant differences were found between the AI treatment and the other treatments (*p* < 0.05). AI showed higher EPS, biofilm C, intracellular P, *Chl‐a*, bacteria and P entrapment in the biofilm. APA, C:P molar ratio in the biofilm, and extracellular P accumulation were the variables contributing less to the effect of DOC bioavailability. Control treatments (AC and BC) did not differ between *before* and *after* phases, making these phases comparable (PERMANOVA, *p* = n.s.). Control treatments were characterised by higher P_intra_/P_extra_ ratio, lower P accumulation in the biofilm, and lower extracellular P.

### 3.2 Effects of Bioavailable DOC on the Linkages Between P‐Related Mechanisms and Biofilm Attributes

**FIGURE 6:**
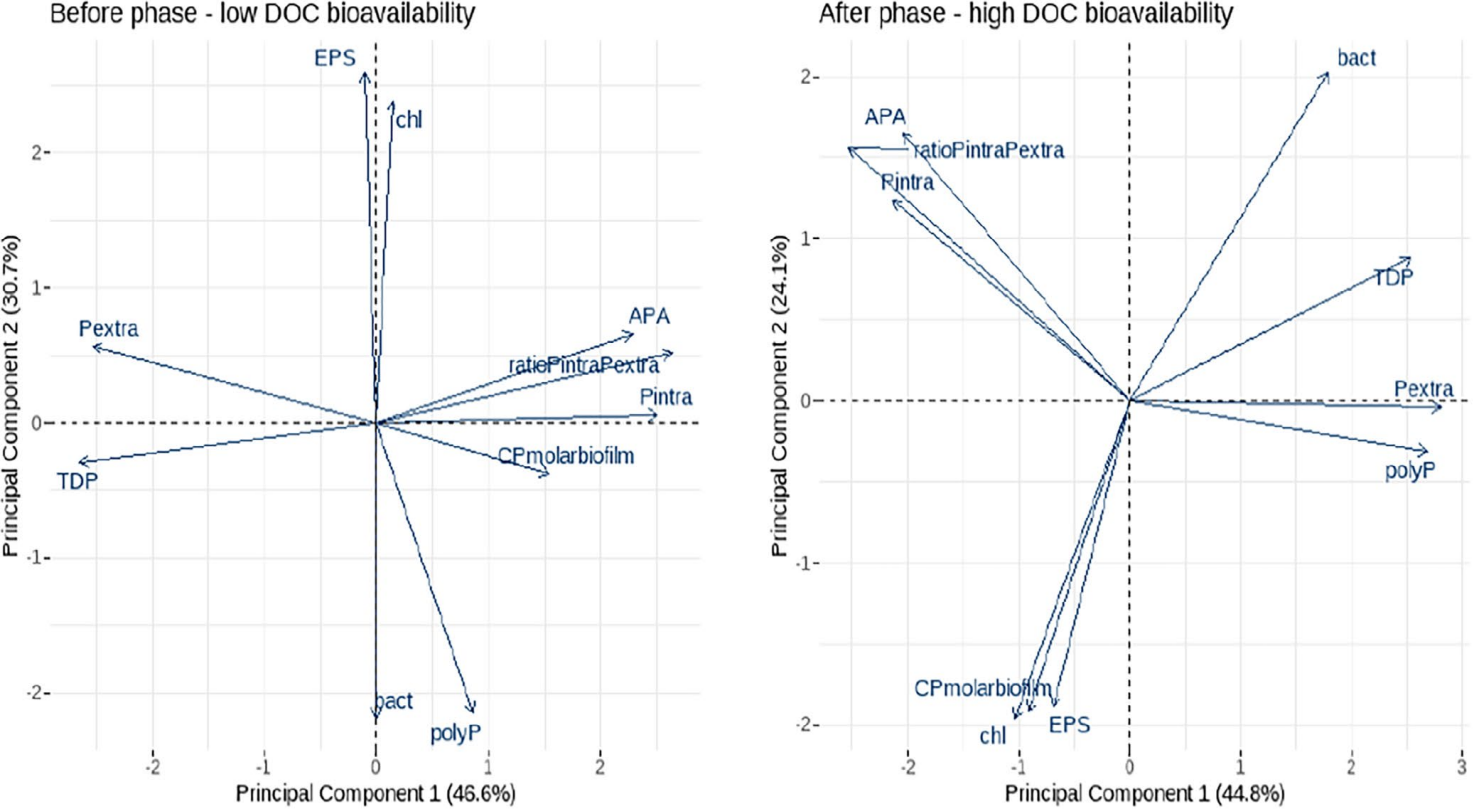
Principal component analysis (PCA) to study the linkage between biofilm variables. The multivariate analysis shows how biofilm variables distribute and interrelate differently under conditions of low DOC bioavailability (left plot) and high DOC bioavailability (right plot). See caption in Figure 5 for abbreviation's description.

To study how the linkage between biofilm structural components, elemental composition, intracellular and extracellular P entrapment and mechanisms of P‐regulation are influenced by DOC bioavailability, we separately analysed the biofilm's multivariate response for the *before* and *after* phases in two PCAs (Figure 6).

In the *before* phase, the PCA accounted for 77.6% of the overall explained variance (Figure 6, left). PC1 explained 46.6% and PC2 explained 30.7%. Under conditions of low bioavailable DOC, P entrapment in the biofilm was strongly influenced by TDP concentrations. At low TDP concentrations (right part of the PCA), intracellular P entrapment dominated, and it was positively linked to higher APA. When increasing TDP concentrations (left part of the PCA), APA, intracellular P, and the ratio P_intra_/P_extra_ decreased, but the biofilm's P entrapment efficiency increased (lower C:P molar ratio) mainly linked to higher extracellular P entrapment. EPS, *Chl‐a*, bacteria density and polyphosphate accumulation were uncorrelated to P entrapment in the biofilm.

In the *after* phase, the PCA accounted for 68.9% of the overall explained variance (Figure 6, right). PC1 explained 44.8% and PC2 explained 24.1%. Under conditions of high bioavailable DOC, the orthogonality between TDP concentration and intracellular P uptake indicates that these two variables are not correlated, in contrast to what was observed under conditions of low bioavailable DOC. Extracellular P entrapment, however, did maintain this linearity with TDP concentrations, increasing as TDP concentration increased. The efficiency of P entrapment in the biofilm (C:P molar ratio) in situations of high DOC bioavailability was not attributed to a specific mechanism of P entrapment but rather to the combined roles of intracellular and extracellular P entrapment. The results showed a strong relationship between bacterial density and the efficiency of P entrapment in the biofilm (C:P molar ratio), although bacterial density was not correlated to a specific P entrapment pathway. EPS and *Chl‐a* were also not correlated to either intracellular or extracellular P entrapment. In both phases, *before* and *after*, we found a positive correlation between EPS and *Chl‐a*, and a negative correlation between *Chl‐a* and bacteria density suggesting some kind of interspecific competition.

## 4 Discussion

### 4.1 Dynamics of Intracellular P Uptake in Stream Biofilms: The Role of DOC Bioavailability

Our findings demonstrate how intracellular P entrapment pathways in stream biofilms are influenced by DOC bioavailability. In conditions of low DOC bioavailability, intracellular P uptake decreased as the TDP concentration increased in the environment. This aligns with observations by Li et al. (2024), who found that in monocultures of both autotrophic and heterotrophic microorganisms, the biomass accrual per unit of P utilisation was higher under low P conditions, suggesting that a nutrient imbalance stimulates the efficiency of P utilisation. Our study corroborated this in mixed microbial communities of fluvial biofilms. Conversely, under conditions of high DOC bioavailability, intracellular P uptake increased at TDP concentrations below 150 μg P/L, but decreased at higher TDP concentrations. The different response on intracellular P uptake between low and high DOC bioavailability could indicate that in river zones with low DOC bioavailability, the capacity for intracellular P uptake in the biofilm is compromised compared to areas with high DOC bioavailability. The carrying capacity of the biofilm, linked to its growth potential (Romani 2010), could explain this phenomenon. Accordingly, biofilms in ecosystems with scarce or absent bioavailable carbon sources may exhibit reduced biomass due to constraints on heterotrophic microbial growth (Soong et al. 2020) and thus could presumably be limited in their capacity for intracellular P uptake.

The fact that intracellular P uptake capacity decreased at certain TDP concentrations, in both scenarios, low and high DOC bioavailability, may be due to a decrease in the efficiency of intracellular P uptake at high TDP. Indeed, P uptake capacity in stream biofilms rapidly declines in response to prolonged high TDP inputs (Price and Carrick 2014), consistent with findings by Lu et al. (2016), who reported a reduction in TDP removal rates as P concentrations in epilithic biofilms increased. This corresponded with the positive relationship between intracellular uptake and APA observed in our study. This indicates that as P limitation decreased (reduced APA), so did the biofilm's capacity for intracellular P uptake. In other words, biofilms in P‐limited systems would exhibit a higher capacity for intracellular P uptake.

The lack of correlation between algal biomass or bacterial density and intracellular P uptake can be explained by the great variability in C:P ratios for bacteria (Kirchman 1994) and phytoplankton (Diehl et al. 2005), demonstrating cell flexibility to capture more or less P depending on environmental characteristics, in this case, DOC bioavailability and TDP concentration. Therefore, intracellular P uptake did not depend on the number of cells in the biofilm but on the capacity of each cell to capture P. The relationship between the molar C:P ratio in the biofilm and different components provides insights into the efficiency of P capture in the biofilm under scenarios of low and high DOC bioavailability. Interestingly, our results showed that under conditions of low bioavailable DOC, the efficiency of P entrapment in the biofilm (i.e., low molar C:P ratio) was closely linked to the extracellular compartment. However, under conditions of high bioavailable DOC, the efficiency of P entrapment in the biofilm (i.e., low molar C:P ratio) was related to bacterial density. Although bacterial density could not be directly linked to intracellular uptake, it suggested that under conditions of high DOC bioavailability, the heterotrophic compartment of the biofilms played a key role in the efficiency of P entrapment. In other words, with high DOC bioavailability, the biofilm is capable of capturing larger amounts of P. High P content in bacterial cells may explain this high P entrapment (Kirchman 1994).

### 4.2 Influence of TDP on Extracellular P Entrapment in Stream Biofilms

Our observations indicate that the extracellular P entrapment in stream biofilms is independent of intracellular P uptake and DOC bioavailability, but strongly influenced by TDP concentration. We noted that as TDP concentrations increase, extracellular P entrapment also increases without reaching a saturation point. In agreement with our results, Tan et al. (2023) found the phosphate adsorption capacity of EPS in algal microaggregates to increase with the initial phosphate concentration. After fitting the P adsorption isotherms of EPS to the Freundlich model, they found a saturation of the adsorption capacity of EPS at phosphate concentrations above 4 mg P/L. Given that this concentration is an order of magnitude higher than those in our experimental flumes, it explains why our findings do not exhibit saturation in the P adsorption capacity of EPS. Our results hence highlight that the capability of extracellular P entrapment in the EPS of fluvial biofilms far surpassed intracellular P uptake and remained unaffected by DOC bioavailability. Yet, the dynamics of P release, and whether they are slower or faster in scenarios of enhanced extracellular P entrapment, require further investigation.

Intriguingly, we did not observe a significant correlation between the concentration of extracellular P and EPS, suggesting that the capacity for extracellular P entrapment does not depend on the quantity of EPS. This is in contrast to the findings of Duan et al. (2023), who noted a strong correlation between EPS content and extracellular P entrapment in laboratory cultures of cyanobacterial aggregates. This discrepancy may be related to the different EPS fractions found in biofilms, namely soluble EPS, loosely bound EPS, and tightly bound EPS. The soluble fraction of EPS typically exhibits weak adhesion to cells. In contrast, the tightly‐bound EPS fraction has been associated with the most stable fraction, directly linked to the surface of bacterial cells and crucial for biofilm structure (Sheng et al. 2010). In biofilm reactors used for wastewater treatment, this tightly‐bound fraction accounts for approximately 30% of the total EPS (Mielcarek et al. 2020). The proportions of the different EPS fractions in fluvial biofilms are unknown; however, possibly only the tightly‐bound EPS fraction plays a key role in the extracellular P entrapment. Furthermore, we measured carbohydrate concentrations within EPS and not proteins, which may explain the observed lack of correlation with extracellular P entrapment. Studies such as those by Schmidt et al. (2016) and Xu, Wu, et al. (2020) have highlighted the critical role of proteins in the adhesive capacity of EPS matrices in biofilms. In particular, positively charged quaternary amines (NH_4_^+^) and tertiary amines (NH_3_^+^), which are common functional groups in proteins, form complexes with negatively charged phosphate (Zhou et al. 2017). Thus, further investigation into the specific roles of different EPS fractions and their protein content in P entrapment is warranted.

### 4.3 Bioavailable DOC Controlled Autotrophic‐Heterotrophic Biomass and P‐Regulation Mechanisms Within Fluvial Biofilms

Our study found that high bioavailable DOC increased bacterial density and sustained the EPS production across the TDP gradient. Specifically, in the presence of bioavailable DOC, EPS concentration remained stable across the TDP gradient, suggesting that DOC bioavailability, rather than nutrient concentration, is the primary driver of EPS production. This indicates that maintaining high DOC bioavailability ensures consistent EPS production regardless of nutrient levels. These findings align with previous research, which has shown that EPS production increases under conditions where bacterial growth is extended by high glucose concentrations in water (Czaczyk and Myszka 2007; Li et al. 2024). Important to notice also is the close coupling between autotrophic carbon production and EPS, as described by Barranguet et al. (2005) and reinforced by the positive correlation found between chl‐a and EPS in our study. In phosphorus‐limited and light‐present scenarios, algae may fix carbon not for growth, due to P scarcity, but for EPS production. This P limitation, accentuated by high DOC bioavailability and a potential competitive interaction between algae and bacteria (see next paragraph), was further underscored by sustained APA within the biofilm in our study. Conversely, our results show that at low bioavailable DOC, EPS production was linked to TDP concentrations. In line with this, earlier research has reported that biofilms at low TDP concentrations are characterised by high polysaccharide concentrations in EPS, whereas at P‐enriched conditions, polysaccharide concentration in EPS decreases (Desmond et al. 2018). The effects of bioavailable DOC reported on bacterial density were expected since the addition of labile DOC causes an augmentation of heterotrophic biomass in fluvial biofilms (Ylla et al. 2012). Similarly, we anticipated no changes in C*hl‐a*, as it primarily depends on light conditions (Jarvie et al. 2002), which were unaltered in our study. The enhanced bacterial density and higher EPS under high bioavailable DOC resulted in increased biofilm biomass and greater accumulation of carbon, nitrogen, and phosphorus in our study.

The observed negative relationship between algae and bacteria, indicated by a negative correlation between chl‐a and bacterial density, suggests a competitive interaction between these two components in the investigated biofilms. Heterotrophs have been shown to outcompete algae for available nutrients in the absence of carbon limitation in aquatic biofilms (Wyatt et al. 2019). Conversely, bacteria are not able to outcompete algae when algae are their sole carbon source (Mindl et al. 2005). Li et al. (2024) demonstrated in a closed mesocosm experiment that competition between bacteria and cyanobacteria under conditions of bioavailable DOC provides a competitive advantage to the heterotrophic component, inhibiting the growth rate of cyanobacteria by 98%. However, this advantage was transient, lasting only 24 h while resources were available. Our study, conducted under semi‐natural experimental conditions using a flow‐through system, suggested that the effects of bioavailable DOC promoting bacterial density can persist for weeks. In scenarios of eutrophication, this dynamic could potentially serve as a control measure for algal growth. This holds true for environments with light levels similar to those reported in our study, as higher light levels could promote a positive effect of algae by stimulating bacterial growth (Ylla et al. 2009), provided no other bioavailable carbon source is available for bacteria (Klug 2005). Regarding the substrate for biofilm colonisation, these findings are generalisable to sandy substrates. On hard substrates (e.g., rocks), a different dynamic between algae and bacteria may occur due to lower organic matter accumulation and the presence of a more autotrophic biofilm (Mora‐Gómez et al. 2016).

The two P‐regulation mechanisms studied (i.e., polyphosphate accumulation and APA) were influenced by DOC bioavailability. In contexts of low bioavailable DOC, polyphosphate levels remained stable across the TDP gradient. This might indicate that the system had reached its maximum uptake capacity in terms of intracellular P uptake. Therefore, there may be no need to adjust polyphosphate levels to capture intracellular P or to increase storage P levels. Furthermore, the lack of a need to develop mechanisms for additional P acquisition (i.e., polyphosphates) could be associated with the decrease in APA along the TDP gradient, which indicates a reduction in P limitation in the biofilm (Chróst and Overbeck 1987). In situations of high bioavailable DOC, polyphosphate acted as a complementary mechanism to low intracellular P uptake, especially at both low and high TDP concentrations. Polyphosphate is a non‐competitive inhibitor of P uptake (Rhee 1973; Price and Carrick 2014), as evidenced by the negative correlation between intracellular P and polyphosphate occurrence in our experiment. This need to complement P uptake is reflected in the sustained levels of APA activity along the TDP gradient, indicating that despite increases in TDP concentration, P limitation persists in systems with high bioavailable DOC. The induction of phosphatase enzyme activity by biofilms can enhance the utilisation and recycling of organic phosphorus in river ecosystems, as previously demonstrated in artificial wetlands (Wu et al. 2018). All of this would be related to the greater P entrapment in the biofilms of systems with high bioavailable DOC, which would confer upon them a higher critical load of phosphorus.

## 5 Conclusions

Bioavailable DOC critically influenced P entrapment in fluvial biofilms. Ecosystems with low bioavailable DOC faced lower biofilm's biomass and constrained bacterial density and resulted in lower P entrapment in biofilms and lower APA. In stark contrast, we found that high bioavailable DOC enhanced biofilm's biomass and bacterial density, thus promoting intracellular P entrapment and higher APA.

The implications of DOC bioavailability on biofilm P dynamics extend into several critical areas of ecosystem functioning. Enhanced P uptake capabilities in systems with high DOC may substantially augment their contribution to river self‐purification processes. Yet the final fate of P in aquatic ecosystems remains to be studied within the perspective of bioavailable DOC. Moreover, the increase in bioavailable DOC fosters bacterial density in river biofilms, which may help to control algal proliferation through competitive resource utilisation. Furthermore, the enhanced ability to capture intracellular P not only stabilises internal nutrient levels but also may contribute to the long‐term entrapment of P, crucial in preventing nutrient overflow into water bodies. Additionally, higher levels of APA activity, driven by high DOC bioavailability, play a pivotal role in recycling dissolved organic phosphorus components. This recycling is vital to prevent the accumulation of these components in the benthic ecosystem, particularly during periods of low water flow and high temperatures, which could exacerbate internal P loading and potentially trigger harmful algal blooms. Conversely, ecosystems with low DOC bioavailability may be more vulnerable to the effects of high TDP concentrations due to their reduced capacity for P entrapment in the biofilm. In these systems, if environmental conditions become favourable, the autotrophic component may dominate unchecked due to the absence of regulatory influence from the heterotrophic microbial component. Such a scenario may lead to the accumulation of dissolved organic phosphorus components, poised for mineralisation under low‐flow and high‐temperature conditions, thereby contributing to internal P loading. All of these factors together may have implications for the eutrophication of aquatic ecosystems. In conclusion, DOC bioavailability seems to be a decisive factor in regulating ecosystem functions, especially in benthic P cycling. This suggests that DOC bioavailability should be considered when understanding and addressing P‐related water quality changes through natural biotic mechanisms.

## Author Contributions

**Nuria Perujo:** conceptualization, methodology, formal analysis, investigation, writing – original draft, writing – review and editing, visualization, supervision, project administration. **Daniel Graeber:** conceptualization, methodology, formal analysis, investigation, writing – review and editing, project administration. **Patrick Fink:** conceptualization, methodology, resources, investigation, writing – review and editing, visualization, project administration. **Lola Neuert:** investigation, methodology, writing – review and editing. **Nergui Sunjidmaa:** methodology, writing – review and editing. **Markus Weitere:** conceptualization, writing – review and editing, project administration.

## Consent

The authors state that the content and authorship of the submitted manuscript have been approved by all authors. All prevailing local, national and international regulations and conventions, and normal scientific ethical practices, have been respected, and consent is given for publication in EMI reports, if accepted.

## Conflicts of Interest

The authors declare no conflicts of interest.

## Supporting information


**Table S1.** Fitting of the quadratic models *y* = ax^2^ + bx + c. Regression models with significant fitting are marked in bold based on a *t*‐statistic > 2, and a *p* value < 0.05 (*p* value < 0.1, marked in italics).

## Data Availability

The data that supports the findings of this study are available in the Supporting Information of this article.
