# Supplementary material for: Bioavailable Dissolved Organic Carbon Serves as a Key Regulator of Phosphorus Dynamics in Stream Biofilms
Source: Environ Microbiol Rep. 2025 Jun 1;17(3):e70115. doi: 10.1111/1758-2229.70115 (PMC12127101; doi:10.1111/1758-2229.70115)
Supplement: Supplementary file 1 — Table S1. Fitting of the quadratic models y = ax2 + bx + c. Regression models with significant fitting are marked in bold based on a t‐statistic > 2, and a p value < 0.05 (p value < 0.1, marked in italics). [file EMI4-17-e70115-s001.docx]

*Supplementary information*

**Bioavailable Dissolved Organic Carbon serves as a Key Regulator of Phosphorus Dynamics in Stream Biofilms**

Nuria Perujo^1^ (nuria.perujo-buxeda@ufz.de)*, Daniel Graeber^2^ (daniel.graeber@ufz.de), Patrick Fink^1,2^ (patrick.fink@ufz.de), Lola Neuert^1^ (lola.neuert@gmail.com), Nergui Sunjidmaa^2^ (nergui.sunjidmaa@ufz.de) , Markus Weitere^1^ (markus.weitere@ufz.de)

^1^Department of River Ecology, Helmholtz Centre for Environmental Research–UFZ, Magdeburg, Germany

^2^Department of Aquatic Ecosystem Analysis, Helmholtz Centre for Environmental Research − UFZ, Magdeburg, Germany

*corresponding author. <https://orcid.org/0000-0002-2072-9283>

Table S1 Fitting of the quadratic models y = ax^2^ + bx + c. Regression models with significant fitting are marked in bold based on a t-statistic >2, and a p-value < 0.05 ( p-value < 0.1, marked in italics).

|  |  | Low bioavailable DOC | | | High bioavailable DOC | | |
| --- | --- | --- | --- | --- | --- | --- | --- |
|  |  | Estimate | Statistic | p-value | Estimate | Statistic | p-value |
| Y = Bact dens.  X = TDP | Intercept  Linear term  Quadratic term | 7.52  3.70  -6.19 | 7.63  1.19  -1.98 | p< 0.001  p = n.s  *p< 0.1* | 55.7  89.6  -38.9 | 13.6  **6.92**  **-3.00** | p< 0.001  **p< 0.001**  **p< 0.05** |
| Y = chl-a  X = TDP | Intercept  Linear term  Quadratic term | 9.69  0.260  5.98 | 8.62  0.073  1.68 | p< 0.001  p = n.s  p = n.s | 14.2  -4.58  0.46 | 4.24  -0.43  0.04 | p< 0.01  p = n.s  p = n.s |
| Y = EPS  X = TDP | Intercept  Linear term  Quadratic term | 49.5  -32.3  57.9 | 6.43  -1.32  **2.38** | p< 0.001  p = n.s  **p< 0.05** | 117  21.1  -52.6 | 4.84  0.276  -0.687 | p< 0.001  p = n.s  p = n.s |
| Y = C biofilm  X = TDP | Intercept  Linear term  Quadratic term | 225  -61.5  11.6 | 8.36  -0.721  0.136 | p< 0.001  p = n.s  p = n.s | 565  476  -512 | 5.86  1.56  -1.68 | p< 0.001  p = n.s  p = n.s |
| Y = N biofilm  X = TDP | Intercept  Linear term  Quadratic term | 33.5  -2.77  -0.252 | 7.98  -0.209  -0.019 | p< 0.001  p = n.s  p = n.s | 80.1  55.5  -67.3 | 7.96  1.74  **-2.12** | p< 0.001  p = n.s  *p< 0.1* |
| Y = P biofilm  X = TDP | Intercept  Linear term  Quadratic term | 2.27  1.97  -0.299 | 12.6  **3.46**  -0.525 | p< 0.001  **p< 0.05**  p = n.s | 4.21  3.75  -2.27 | 13.0  **3.65**  **-2.21** | p< 0.001  **p< 0.01**  *p< 0.1* |
| Y = C:P molar biofilm  X = TDP | Intercept  Linear term  Quadratic term | 41.5  -29.7  3.38 | 5.59  -1.26  0.144 | p< 0.001  p = n.s  p = n.s | 53.4  -4.82  -14.6 | 5.33  -0.152  -0.462 | p< 0.01  p = n.s  p = n.s |
| Y = P intra  X = TDP | Intercept  Linear term  Quadratic term | 1.17  -0.892  -0.518 | 10.8  **-2.59**  -1.51 | p< 0.001  **p< 0.05**  p = n.s | 2.72  -0.207  -3.13 | 10.2  -0.245  **-3.70** | p< 0.001  p = n.s  **p< 0.01** |
| Y = P extra  X = TDP | Intercept  Linear term  Quadratic term | 1.10  2.87  0.219 | 11.2  **9.23**  0.706 | p< 0.001  **p< 0.001**  p = n.s | 1.49  3.96  0.855 | 8.95  **7.50**  1.62 | p< 0.001  **p< 0.001**  p = n.s |
| Y = ratio Pintra/Pextra  X = TDP | Intercept  Linear term  Quadratic term | 2.37  -4.17  2.13 | 9.21  **-5.12**  1.62 | p< 0.001  **p< 0.01**  **p< 0.05** | 4.20  -7.81  3.79 | 4.21  **-2.47**  1.20 | p< 0.01  **p< 0.05**  p = n.s |
| Y = APA  X = TDP | Intercept  Linear term  Quadratic term | 0.032  -0.021  0.014 | 18.8  **-3.94**  **2.65** | p< 0.001  **p< 0.01**  **p< 0.05** | 0.036  -0.001  -0.004 | 15.4  -0.19  -0.56 | p< 0.001  p = n.s  p = n.s |
| Y = polyP  X = TDP | Intercept  Linear term  Quadratic term | 5.85  -0.78  0.115 | 27.2  -1.15  0.680 | p< 0.001  p = n.s  p = n.s | 3.43  -0.47  2.52 | 9.78  -0.421  **2.28** | p< 0.001  p = n.s  *p< 0.1* |
